# Differences and potential mechanisms of theta oscillation and temporoparietal and temporal-central networks in temporal lobe epilepsy patients with unilateral hippocampal sclerosis

**DOI:** 10.1186/s42494-024-00170-7

**Published:** 2024-08-25

**Authors:** Chenxi Qiu, Chenxi Zhong, Ying Liu, Liju Wang, Yingying Tang, Zhiyi Liu, Sijia Guo, Yingqi Jiang, Enzhi Li, Jing Lu, Bo Yan, Xiaoting Hao, Dong Zhou

**Affiliations:** 1 MOE Key Lab for Neuroinformation, School of Life Science and Technology, University of Electronic Science and Technology of China, Chengdu , Sichuan, 611731 China; 2 Department of Neurology, West China Hospital, Sichuan University, Chengdu , Sichuan, 610041 China; 3 School of Nursing, Sichuan University, Sichuan, 610041 China; 4 Department of Neurology, Chengdu Shangjin Nanfu Hospital, Chengdu, Sichuan Province 611730 China; 5 School of Public Health, Sichuan University, Chengdu, 610044 China

**Keywords:** Mesial temporal lobe epilepsy, Hippocampal sclerosis, Electroencephalography, Anti-seizure medications, Theta oscillation

## Abstract

**Background:**

There is a lack of further exploration of the epileptogenic network of specific types of epilepsy, such as unilateral hippocampal sclerosis (HS), and there is an urgent need to find exact evidence to confirm the consistency of its brain network changes.

**Methods:**

We enrolled 22 mesial temporal lobe epilepsy with hippocampal sclerosis (mTLE-HS) patients to compare the differences in brain activity between 22 healthy controls (HCs) and them. Resting-state electroencephalography (EEG) was also measured. Then, we calculated the power spectral density and phase locking values in and between these electrodes.

**Results:**

The results showed the increased theta power was related to the high severity of epilepsy in the temporal, parietal, and central regions in mTLE-HS patients, and there were positive correlations between theta power in the contralateral temporal region and seizure frequency. Theta power in the ipsilateral parietal lobe is positively correlated with the number of anti-seizure medications (ASMs), but not with the usage of third-generation ASMs. Meanwhile, the temporal lobe of mTLE-HS patients had more connectivity with parietal lobe and central region.

**Conclusions:**

Theta power is an important EEG indicator of mTLE-HS, positively correlates with epilepsy severity and seizure frequency, and has network properties that can be observed outside the lesion. Moreover, the usage of third-generation ASMs did not affect the risk of increased theta power. Lastly, the temporoparietal and temporal-central networks are likely to be causative pathways in epilepsy patients with cognitive impairment. This study provides a potential guideline for the treatment of mTLE-HS in clinical practice.

**Supplementary Information:**

The online version contains supplementary material available at 10.1186/s42494-024-00170-7.

## Background

Temporal lobe epilepsy (TLE) represents the most prevalent form of focal epilepsy in adults, with the most common histopathological feature: hippocampal sclerosis (HS) [1, 2]. Mesial temporal lobe epilepsy with hippocampal sclerosis (mTLE-HS), a well-defined epileptic syndrome, is prone to be drug-resistant epilepsy (DRE) [3] and can lead to cognitive impairment [4–7], further aggravating disease burden and seriously affecting the quality of life for patients and their families [8].

Electroencephalogram (EEG), as the most important tool for the diagnosis and localization of epilepsy, showed ictal rhythmic activity had a frequency of 4.7 ± 1.5/s (range 1–8/s), which mostly localized in the anterior temporal region [9], and interictal unilateral 5–9 Hz rhythmic theta or alpha epileptiform activity that reached its peak at the anterior temporal scalp electrodes in patients with unilateral mTLE-HS [10]. Previous studies have found the theta power at the ipsilateral hemisphere is higher in patients with focal epilepsy than that in healthy controls (HCs) [11–13]. In patients with TLE, quantitative EEG revealed higher theta power in the contralateral parietal and occipital regions, which showed a correlation with longer epilepsy duration [14]. Intracerebral EEG detected the frequency-power predominance of theta oscillations during interictal recording for most subfields in mTLE-HS patients [15].

Epilepsy has been considered as a neural network disorder involving both generalized and focal epilepsy [16–21]. Resting-state functional magnetic resonance imaging (MRI) demonstrated brain connectivity impairments that extended beyond the epileptic lesion with the involvement of bilateral cortico-subcortical regions in patients with unilateral mTLE-HS [20, 21]. Meanwhile, resting-state EEG detected increased extensive connectivity, particularly involving the right posterior cingulate gyrus in patients with unilateral mTLE-HS [22]. Among these patients, increased theta power was found to be associated with stronger functional connectivity, suggesting that functional networks may serve as reliable predictors of the effects across each frequency in mTLE-HS patients [23]. Additionally, reinforced theta connectivity in the ipsilateral hemisphere was also observed in mTLE-HS patients [24] and was further correlated with attention, memory, and language impairments [23, 25, 26].

However, the brain regions in which abnormal interictal theta activity occurs in mTLE-HS patients and the pathologic correlates of these abnormal findings are largely unexplored. Hence, the primary objective of this study is to comprehensively examine interictal EEG theta activity in mTLE-HS patients and establish correlations between the characteristics of theta rhythms and clinicodemographic variables. We have quantified the theta, alpha, and beta power and functional connectivity in both mTLE-HS patients and HCs, to investigate whether there are globally and/or localized changes in interictal theta background activity in mTLE-HS patients. Additionally, we examined correlations between theta power and clinical indicators to assess the relationship of abnormal theta activity with seizure factors and medication usage. We hypothesized that a combined analysis of EEG and clinical data measures can provide improved guidance for the clinical diagnosis and treatment of mTLE-HS patients.

## Methods

This cohort study was approved by the Ethics Committee of Sichuan University (No. K2021037), and informed consent was obtained from all patients.

Patients were enrolled in the Epilepsy Center of West China Hospital, Sichuan University (Chengdu, China) from Mar. 2023 to Sept. 2023. Inclusion criteria: (1) patients whose diagnosis made by trained epileptologists based on clinical symptoms, neuroimaging (MRI or PET/MR in some cases) and electroencephalography (EEG) or video electroencephalography (VEEG) monitoring data of temporal lobe epilepsy with hippocampal sclerosis according to the ILAE criteria [27]; (2) age of 18–60 years; and (3) voluntarily providing informed consent to participate. Exclusion criteria: (1) presence of other major lesions revealed by MRI; (2) with bilateral hippocampal sclerosis; (3) occurrence of seizures within 24 h before the EEG acquisition; (4) with a history of mental retardation, alcohol or drug abuse, or uncontrolled psychosis; and (5) contradiction or intolerability to EEG recording. Based on a comprehensive evaluation of semiology, EEG, and MRI findings, patients were divided into two groups: left HS group (HS-L, *n* = 11,) and right HS group (HS-R, *n* = 11).

HCs were recruited from invitation based on the following criteria: (1) age of 18–60 years; (2) no symptoms or history of neurological and psychiatric disorders; (3) no history of head trauma, surgery or substance abuse; and (4) voluntarily providing informed consent to participate in the trial.

### Clinical information definition

Patients were classified into two groups: drug-resistant epilepsy (DRE) and non-DRE, based on their seizure control. DRE was defined as the inability to achieve sustained seizure freedom despite receiving adequate and tolerable treatment with at least two appropriate anti-seizure medications (ASMs), either as monotherapy or in combination, for a sufficient duration [28]. Seizure freedom was defined as the absence of seizures for at least 12 months or three times the pretreatment inter-seizure interval, whichever was longer, at the time of assessment [28]. The study focused on third-generation ASMs, which are ASMs launched after 2000 [29]. The specific ASMs involved in this study were zonisamide (ZNS), lacosamide (LCM), and perampanel (PER). Seizure severity was evaluated using The National Hospital Seizure Severity Scale (NHS3) [30], with higher scores indicating more severe episodes.

### EEG data acquisition

Scalp EEG data were obtained from participants while they were in a quiet, and relaxed state with their eyes open. To ensure a high-quality recording, noise-canceling headphones were used. The EEG signals were collected using a 32-channel instrumented EEG-1200C electroencephalograph (Tokyo, Japan) based on an international 10–20 system of electrode placement (several extra electrodes were utilized, including Fpz, FC3, FC4, FCz, and Oz). Fpz is positioned at the midpoint between Fp1 and Fp2, FCz is located at the midpoint between Fz and Cz, FC3 and FC4 are located at the 10% position to the left and right of FCz respectively, and Oz is situated at the midpoint between O1 and O2. The EEG data were sampled at a rate of 500 Hz. Refering to previous similar studies [14, 15], continuous recordings were performed for 8 min.

### EEG data processing

Data were preprocessed using the Brainstorm toolbox (http://neuroimage.usc.edu/brainstorm) in MATLAB 2016b (Natick, MA: MathWorks). The 24-channel EEG data were initially filtered using the bandpass Finite Impulse Response (FIR) filter with a range of 0.5–100 Hz. Additionally, the IIR Notch filter at 50 Hz followed by Independent Component Analysis (ICA) was applied to remove eye movement artifacts. Re-referencing was performed to the average value of all the channels. For the HS-R group, a horizontal contralateral flip was applied to the EEG channels. This means that the left side reflected the ipsilateral side of the HS, while the right side reflected the contralateral side of the HS. A proportional selection of the HCs group also underwent the horizontal flip for consistency. The alpha (8–13 Hz), beta (14–30 Hz) and theta (4–7 Hz) frequency bands were extracted from the EEG data using the fast Fourier transformed (FFT). Power spectral density (PSD) was then calculated using Welch’s method in these three frequencies ranges. For further analysis, specific electrodes with significant PSD changes (including T3, T4, P3, C3, C4, Cz, and FC4) were selected as regions of interest (ROI). The correlation between theta power and clinical characteristics was computed using these ROIs. The ROI-to-ROI phase-locking value (PLV) was calculated as the following formula:

$$\mathrm{zi}\left(t\right)=\mathrm{si}\left(\mathrm t\right)+\mathrm{jHT}\left(\mathrm{si}\left(t\right)\right)$$

where ${\text{si}}({{t}})$ represents signal, ${\text{zi}}({{t}})$ represents the signal after Hilbert transformation. Then, the phase difference ($\triangle \Phi (t)$) was calculated between the signals:

$$\triangle\Phi\left(t\right)=\arg\left(\frac{z1\left(t\right)z2\ast\left(t\right)}{\left|z1\left(t\right)\vert z2\left(t\right)\right|}\right)$$

Lastly, PLV was calculated according to the equation:

$$\text{PLV} (t) = \left| \text{E} \left[ e^{j \triangle \Phi (t)} \right] \right|$$

### Statistical analysis

IBM SPSS 20.0 was utilized to perform the statistical analysis. Numerical variables were presented as means (standard deviation, SD) or median (range), while categorical variables were described as numbers. The Chi-square (χ^2^) test was used for categorical variables, and the Student’s *t*-test or Mann–Whitney test was employed for continuous variables according to their normal distribution and variance homogeneity. The power spectral density and phase-locking value differences between the mTLE-HS patients and HCs were compared. Correlation analysis (including Pearson's, Spearman, or point biserial correlation analysis) was performed to explore the relationship between the significant PSD in ROI and clinical characteristics (such as age, epilepsy duration, drug resistance, seizure freedom, seizure frequency, number of ASMs, third-generation ASMs, and NHS3 score). To further investigate the potential influence of third-generation ASMs on epilepsy duration, seizure frequency, and NHS3 score, a comparison was made between the mTLE-HS patients treated with third-generation ASMs (3G ASMs, *n* = 8) and those without (non-3G ASM, *n* = 14), aiming to ascertain if any differences in these three factors existed between the two subgroups and to rule out the excessively high values of these factors in the non-3G ASM group may have influenced the elevated theta power, thus resulting in no correlation between third-generation ASMs and theta power. A *P*-value < 0.05 was considered statistically significant.

## Results

A total of 22 patients (10 males and 12 females) were enrolled in the study, with the median age of 30.0 years (interquartile range: 24.5–39.3 years). Meanwhile, the healthy control group consisted of 22 individuals (14 males and 8 females) with a median age of 39.7 years (interquartile range: 25.8–46.0 years). There were no statistically significant differences in terms of sex and age between the two groups. In the mTLE-HS patient group, the mean age of epilepsy onset was 19.6 ± 11.7 years. The mean duration of epilepsy was 12.5 ± 10.3 years, and the median seizure frequency in the most recent year was 12 days/year (interquartile range: 6–32.3 days/year). Among these mTLE-HS patients, 22.7% had a history of febrile seizures, 31.8% had experienced status epilepticus, and a half had DRE. Only 13.6% of them achieved seizure freedom with medication, and the mean NHS3 score was 8.73 ± 2.55. In terms of treatment, 54.5% of them received biotherapy as the predominant treatment approach, whereas 36.4% receive third generation ASMs. A summary of the clinicodemographic characteristics of the mTLE-HS group and the HCs group is presented in Table 1.

**Table 1:** Clinicodemographic characteristics of the mTLE-HS group and the HCs group

Characteristics	mTLE-HS group *n* = 22	HCs group *n* = 22	*P *value
**Male, ***n*** (%)**	10(45.5)	14(63.6)	0.226
**Age, years, median (IQR)**	30.0(24.5–39.3)	39.7(25.8–46.0)	0.107
**Age at epilepsy onset, years, mean ± SD**	19.6 ± 11.7	-	-
**Epilepsy duration, year, mean ± SD**	12.5 ± 10.3	-	-
Seizure frequency*,days/year, median (IQR)	12(6–32.3)	-	-
**History of febrile seizure, ***n*** (%)**	7(31.8)	-	-
**History of status epilepticus, ***n*** (%)**	5(22.7)	-	-
**Drug-resistant epilepsy (DER), ***n*** (%)**	11(50.0)	-	-
**Seizure freedom, ***n*** (%)**	3(13.6)	-	-
**NHS3 score, mean ± SD**	8.73 ± 2.55	-	-
**Number of ASMs (currently used), ***n*** (%)**		-	-
** 1**	5(22.7)	-	-
** 2**	12(54.5)	-	-
** 3**	3(13.6)	-	-
** ≥ 4**	2(9.0)	-	-
Third-generation ASMs**,*n*** (%)**	8(36.4)	-	-

*Abbreviation:* *mTLE-HS* mesial temporal lobe epilepsy with hippocampal sclerosis, *HCs* healthy controls, *ASMs* anti-seizure medications, *NHS3* The National Hospital seizure severity scale, *IQR* interquartile range, *SD* standard deviation

Values are *n* (%), median (IQR), and mean ± SD

^*^Seizure frequency in this study refers to the number of seizures in the latest year (data were selected as the maximum value after quantification based on the information filled in by patients), *n* = 20, one case was not available due to unquantifiable data and one case was deleted as an outlier

^**^Third-generation ASMs in this research involve zonisamide (ZNS), lacosamide (LCM), and perampanel (PER)

### PSD in different band frequencies in mTLE-HS patients

**Fig. 1:**
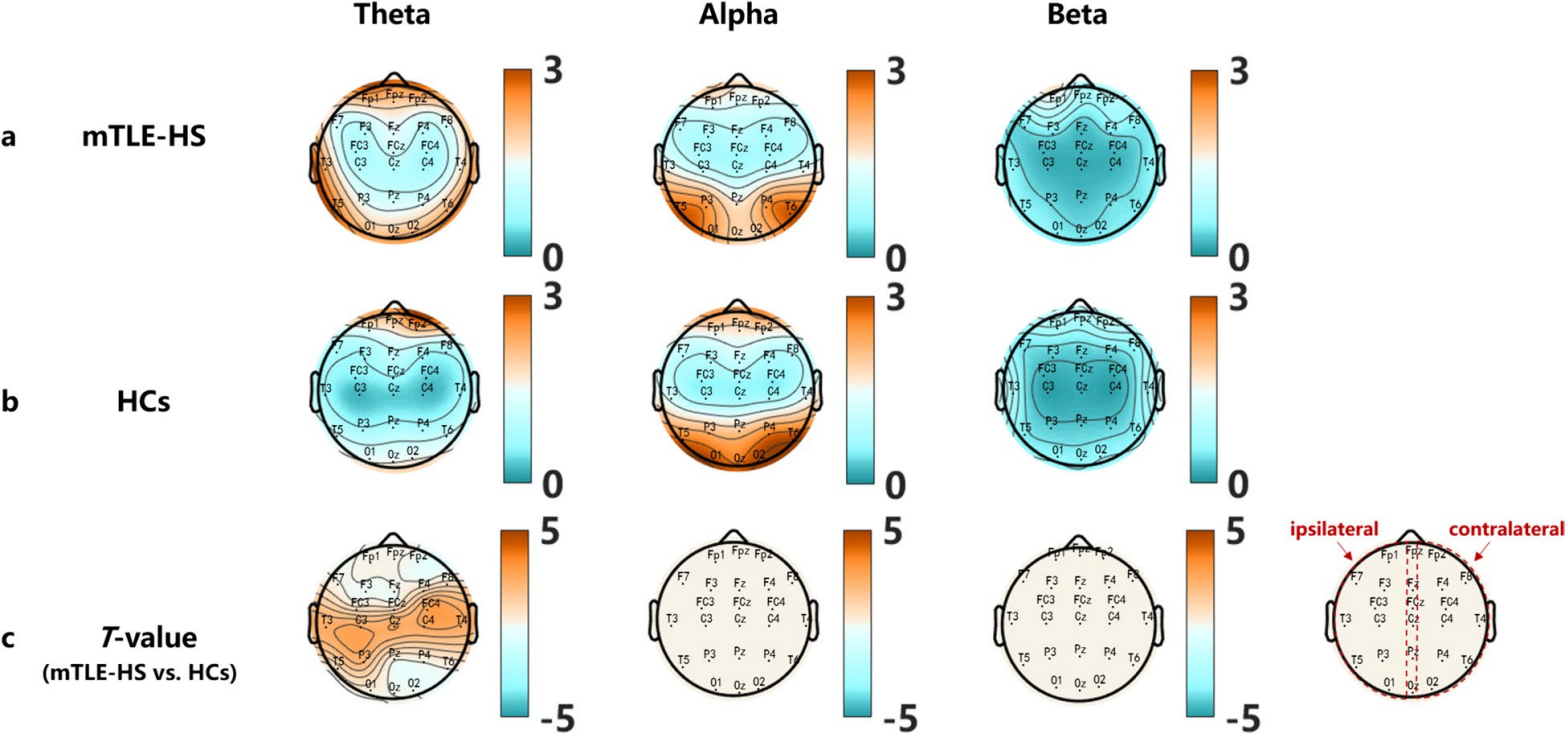
PSD topographical maps of three frequency bands (**a**) the mTLE-HS group, (**b**) the HCs group, (**c**) mTLE-HS *vs.* HCs differences. Orange color indicates mTLE-HS > HCs in the power spectrum, blue color vice versa. *P* < *0.05,* the *T*-value for *P* > *0.05* was set to 0 (white color)

Theta band showed significantly higher PSD in the T3/4, P3, C3/4, Cz, and FC4 electrodes in the mTLE-HS group than the HCs (*T*_*T3*_= *2.32, T*_*T4*_= *2.22, T*_*C3*_= *2.29, T*_*C4*_= *2.79, T*_*Cz*_= *2.40, T*_*P3*_= *2.05, T*_*FC4*_= *2.55, P*<*0.05*). However, no significant increase in PSD was detected in the alpha and beta bands, and no significant PSD decrease was detected in any of the frequency bands when comparing patients with mTLE-HS to HCs Fig. 1.

### The correlation between clinical data and PSD in ROI

**Fig. 2:**
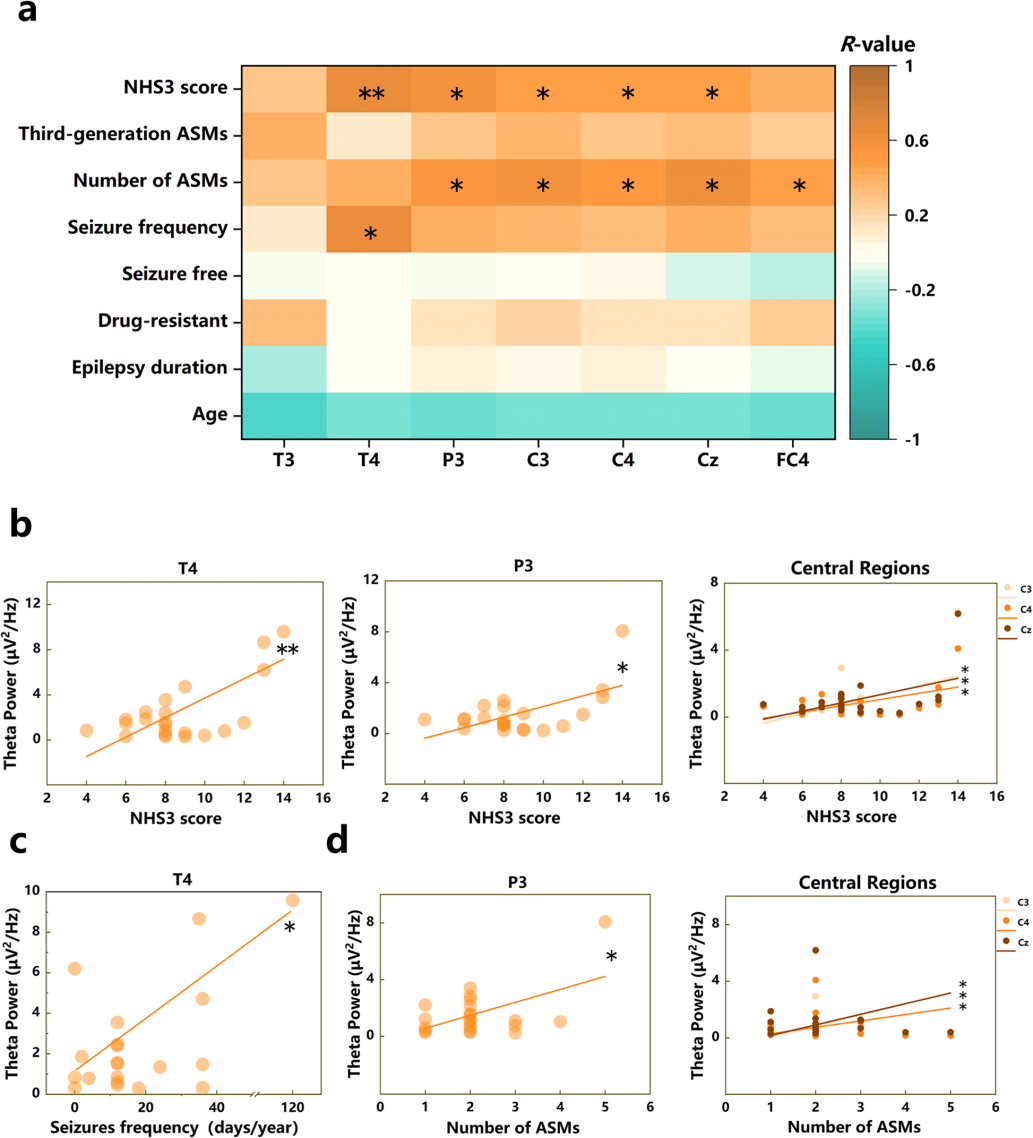
**a** Correlation between PSD in theta band and clinical data in the mTLE-HS group. **b** Correlations between the theta power in P3, the average value of C3/4 and Cz, T4 and the NHS3 scores. **c** Correlations between the theta power in T4 and seizure frequency. **d** Correlations between the theta power in P3, the average value of C3/4 and Cz and the number of ASMs. Only the electrode related to NHS3 scores were shown based on (**a**). *P < 0.05, **P < *0.01*, FDR-corrected

As shown in Table 2 and Fig. 2, the NHS3 score exhibited a positive correlation with the theta power of most of the significant PSD: P3 (*R* = *0.57, P* < *0.05*), T4 (*R* = *0.66, P* < *0.01*), C3 (*R* = *0.47, P* < *0.05*), C4 (*R* = *0.46, P* < *0.05*), and Cz (*R* = *0.49, P* < *0.05*), except T3 (*R* = *0.29, P* = *0.20*) and FC4 (*R* = *0.39, P* = *0.09*). The seizure frequency showed a positive trend related to the theta power in T4 (*R* = *0.64, P* < *0.05*). The number of ASMs showed a positive trend related to the theta power in P3 (*R* = *0.53, P* < *0.05*), C3 (*R* = *0.57, P* < *0.05*), C4 (*R* = *0.52, P* < *0.05*), Cz (*R* = *0.61, P* < *0.05*), and FC4 (*R* = *0.47, P* < *0.05*). Interestingly, there was no significant correlation observed between the theta power and the usage of third-generation ASMs in any of the ROIs (*R*_*T3*_ = *0.40, R*_*T4*_ = *0.09, R*_*P3*_ = *0.29, R*_*C3*_ = *0.36, R*_*C4*_ = *0.30, R*_*Cz*_ = *0.34, R*_*FC4*_ = *0.24, P* > *0.05*).

**Table 2:** Correlation between PSD in theta band and clinical data in the mTLE-HS group

Characteristics	Theta
Age	-
Epilepsy duration	-
Drug-resistant epilepsy (DRE)	-
Seizure freedom	-
Seizure frequency (seizure days/year)	T4^*^(*R* = *0.64*)
Number of ASMs	P3^*^(*R* = *0.53*) C3^*^(*R* = *0.57*) C4^*^(*R* = *0.52*) Cz^*^(*R* = *0.61*) FC4^*^(*R* = *0.47*)
Third-generation ASMs^*a*^	-
NHS3 sores	T4^**^(*R* = *0.66*) P3^*^(*R* = *0.57*) C3^*^(*R* = *0.47*) C4^*^(*R* = *0.46*) Cz^*^(*R* = *0.49*)

*Abbreviation*: *ASMs* anti-seizure medications, *NHS3* The National Hospital Seizure Severity Scale

*a*: Third-generation ASMs refer to anti-seizure medications launched after 2000, and this experiment involves zonisamide (ZNS), lacosamide (LCM), and perampanel (PER)

*P < 0.05,**P < 0.01,FDR-corrected

Furthermore, no significant differences were found in the NHS3 score (*U* = *54, P* = *0.91*), epilepsy duration (*T* = *0.71*, *P* = *0.49*), and seizure frequency (*U* = *23, P* = *0.93*) between the non-3G ASM group and 3G ASMs group. This finding suggests that it is unlikely that the higher values of these three factors in the non-3G ASM group influenced the higher theta power, leading to close theta power levels between the two subgroups, which results in theta power being independent of third-generation ASMs. Therefore, our results indicate that the usage of third-generation ASMs may not have an impact on theta power.

### Comparison of ROI-wise PLV between mTLE-HS groups and HCs

**Fig. 3:**
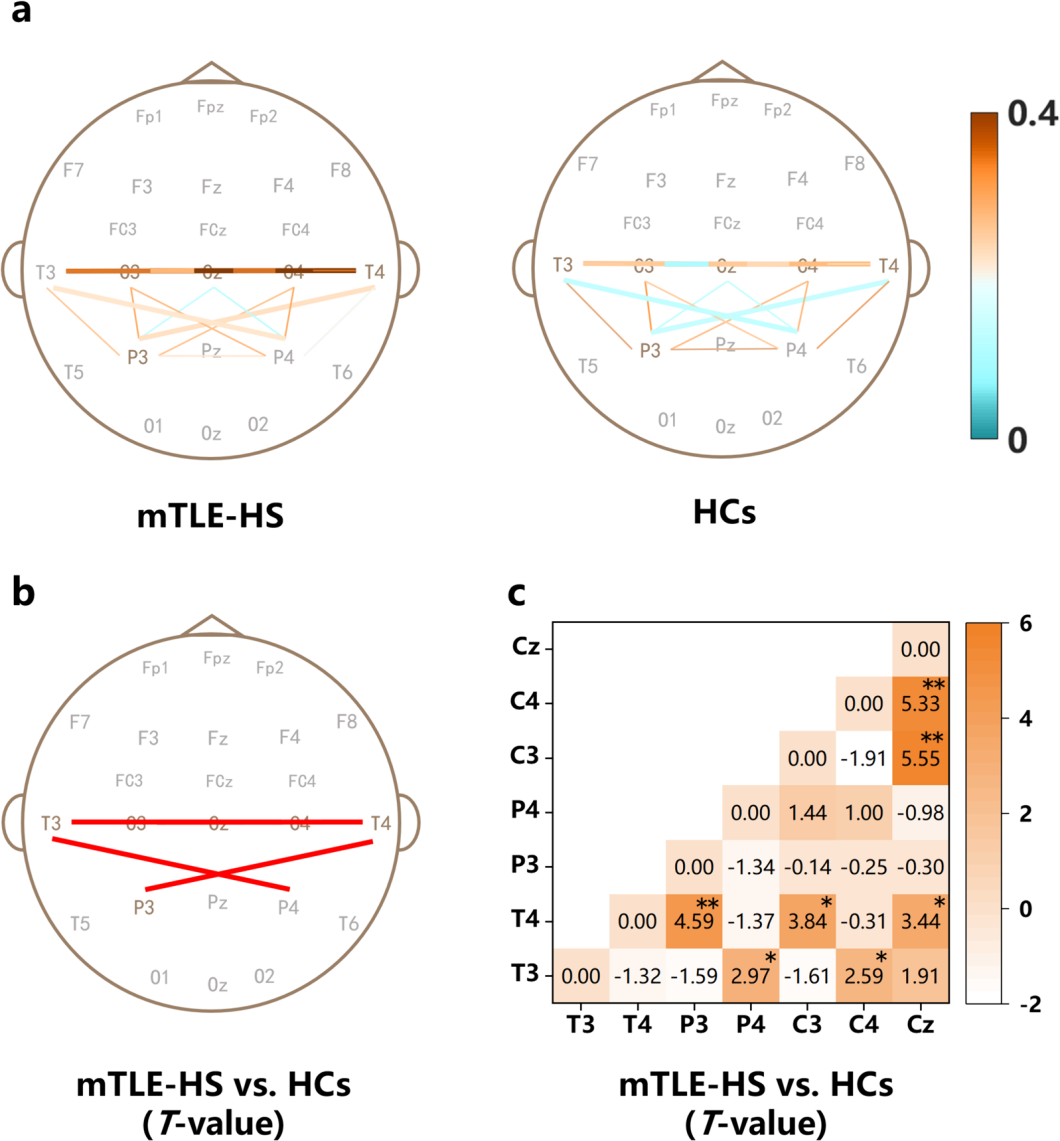
Changes in phase-locking value in theta band. **a** Brain networks for mTLE-HS and HCs in ROI-to-ROI, respectively, thick lines represent significant differences between groups*.* **b**, **c** mTLE-HS *vs.* HCs differences in ROI-to-ROI, the *T*-value for *P* > *0.05* was set to 0. FDR-corrected. **b** Red lines represent mTLE-HS > HCs. **c** Orange represents mTLE-HS > HCs, *P < 0.05, ** P < *0.01*

Based on the significant differences observed in PSD between two groups, the electrodes T3, T4, P3, P4, C3, C4, and Cz were selected as ROIs to calculate the ROI-to-ROI PLV in the theta band for both the mTLE-HS and HCs. As depicted in Fig. 3, compared to the HCs group, the mTLE-HS patients demonstrated significantly increased phase-locking value of theta frequency between T3 and P4 (*T* = *2.97, P* < *0.05*), T3 and C4 (*T* = *2.59, P* < *0.05*), T4 and P3 (*T* = *4.59, P* < *0.01*), T4 and C3 (*T* = *3.84, P* < *0.05*), T4 and Cz (*T* = *3.44, P* < *0.05*), C3 and Cz (*T* = *5.55, P* < *0.01*), and C4 and Cz (*T* = *5.53, P* < *0.01*).

## Discussion

In this study, we observed a significant increase in theta PSD in the bilateral temporal, bilateral central, and ipsilateral parietal regions of mTLE-HS patients compared to HCs. However, there were no significant differences in alpha and beta PSD in the whole brain. The increased theta power in the contralateral temporal lobe was related to higher seizure frequency and NHS3 scores. The increased theta power in the ipsilateral parietal lobe was associated with a higher number of ASMs used and higher NHS3 scores. Notably, the use of third-generation ASMs did not show any association with the increase in theta power. Furthermore, our analysis of ROI-wise phase-locking values in the theta frequency revealed increased functional connectivity between the bilateral temporal lobe and contralateral parietal lobe, as well as with the contralateral central regions in mTLE-HS patients. Additionally, enhanced functional connectivity was observed within the central regions. Our findings supported the importance of theta rhythms in neurophysiological studies of brain networks in mTLE-HS and explored the relationship between theta rhythms and epilepsy severity, between theta rhythms and mechanisms of action of ASMs, as well as third-generation ASMs for the first time.

### Increased *theta* power suggests epilepsy severity in mTLE-HS patients

Our results showed an elevation in theta power and no significant changes in alpha and beta power in the brains of patients with mTLE-HS. These results align with previous studies that have also reported significant increases in theta power abnormalities among patients with focal epilepsy [11–13]. Fonseca et al. found significant incremental changes in theta power abnormalities in patients with TLE [14]. Reyes-garcia et al. also identified higher theta abnormalities specifically in patients with mTLE-HS [15]. Consistent with these studies, we found an increase in theta power without a decrease in patients with mTLE-HS. Moreover, our ROI-wise functional connectivity analysis demonstrated enhanced electrode-to-electrode connections, and previous studies have suggested that this theta increase may be due to time-dependent changes in structural lesions and seizure frequency [31].

Previous studies have found that increased theta power is associated with seizure frequency [32], however, there is limited research focusing on specific brain regions. In our study, we found that higher theta power in contralateral temporal lobe of mTLE-HS patients was associated with a higher seizure frequency. In addition, the NHS3 scores, which can reflect the severity of epilepsy, frontal and occipital theta power has been found to correlate with convulsive seizures in TLE [33]. Furthermore, hippocampal theta power has been associated with seizure duration and automatism [34, 35]. In our study, we found a relationship between increased theta power in the contralateral temporal lobe and high NHS3 scores in mTLE-HS patients. This suggests that the heightened theta power in the contralateral temporal lobe of mTLE-HS patients may reflect the cumulative damage resulting from repeated seizures. Moreover, Fonseca et al. found that higher theta power in the contralateral temporal region was associated with longer epilepsy duration, indicating progressive seizure-related damage in the contralateral temporal region in patients with TLE [14].

### Elevated *theta* power in bilateral temporal, central, and ipsilateral parietal regions in mTLE-HS patients

In mTLE-HS patients, the presence of ictal temporal theta with high localization value has been well described [9]. Additionally, interictal abnormal increases in temporal theta have also been reported [15], involving wider regions including the parietal and central areas [14, 36], with an accuracy of 92.04% in the lateralization [37]. Consistent with previous studies, our quantitative analysis revealed elevated theta PSD not only in the temporal lobe ipsilateral to the lesion, but also in the contralateral temporal lobe, ipsilateral parietal lobe and bilateral central regions in mTLE-HS patients. This is in line with findings in TLE, where theta abnormalities were not confined solely to the ipsilateral side, but also involved the parietal lobe, central region, and occipital lobe bilaterally [14]. As for NHS3 scores, occipital and frontal lobes have been found to be associated with loss and retention of consciousness in parietal and occipital lobe epilepsy [38], without reporting on theta power at that time. In our study, we found that increased theta power in the ipsilateral parietal and central regions was related to NHS3 scores in mTLE-HS patients. This abnormal theta activity in the temporal, parietal, and central regions may be linked to network changes due to abnormal discharges from the hippocampal lesions in mTLE-HS patients [11, 39]. That is, although the extent of lesions in mTLE-HS patients may be limited, the impact of damage may not be limited due to the presence of brain networks.

### Increased *theta* power linked to number of ASMs used, except for the third-generation drugs

Our results found that higher theta power in the ipsilateral parietal and central regions of mTLE-HS patients was associated with a greater number of ASMs used and higher NHS3 scores. Previous studies have also reported that increased theta power in the ipsilateral parietal and central lobes in focal epilepsy is associated with the mechanisms of action of ASMs [36, 40–42]. These findings may be indicative of cognitive deterioration and subjective neurotoxicity associated with increased theta power [36]. On this basis, we found no association between third-generation ASMs and increased theta power in ipsilateral brain regions of mTLE-HS patients. Furthermore, longer duration of epilepsy, higher seizure frequency, and higher NHS3 scores might contribute to elevate theta power in these regions. However, there were no significant differences in these factors between patients who had used third-generation ASMs and those who had not. This excludes the possibility that the higher theta power of patients not using third-generation ASMs is due to several factors, which could make their theta power close to that of patients using the third-generation ASMs, thereby suggesting no correlation between the use of third-generation ASMs and theta power. It might indicate that the participation of ASMs in abnormal theta rhythms in the parietal and central brain regions of mTLE-HS patients, and subsequent effects on executive function and working memory [43], is not related to the use of third-generation drugs such as ZNS, LCM, and PER. These findings may guide clinicians in choosing more appropriate third-generation medications for mTLE-HS patients, especially those with abnormal slow-wave activity and concomitant severe cognitive impairment.

### Higher connectivity in the temporoparietal and temporal-central network in mTLE-HS patients

We found an increase in connectivity between the contralateral temporal lobe and parietal lobe, as well as the central regions in mTLE-HS patients. Additionally, we observed increased connectivity between the bilateral temporal lobe and contralateral parietal lobe, between the bilateral temporal lobe and contralateral central regions, and within the central regions. Studies on the temporoparietal network have shown varying results. Duma et al. found a greater integration index of temporoparietal networks in TLE patients [44]. Fleury et al. reported increased temporoparietal networks in mTLE-HS patients, with increased left and right networks associated with visual memory and verbal memory, respectively [45]. However, Dumlu et al. found that the temporoparietal connectivity decreases in TLE patients [46], which is inconsistent with our results, possibly due to differences in neuroimaging methods and the approach used to compute connections. Regarding the temporal-central and intra-central networks, Maneshi et al. reported increased temporal-central network connectivity in TLE patients [47]. Morgan et al. also reported increased hippocampal-central network connectivity in patients with mTLE-HS, suggesting that the initial seizures may be associated with heightened connectivity in this network [48]. Meanwhile, they found increased intra-central network connectivity in mTLE-HS patients, suggesting that the temporal-central network was increasingly influenced by this network.

### Limitations

This study has several limitations that should be considered. Firstly, the sample size was small. Secondly, the diagnosis of HS relies solely on clinical and imaging criteria, and not all patients ubderwent pathological confirmation, therefore, continued patient follow-up in further studies is needed. Lastly, due to the deep location of the hippocampus and the limited spatial resolution of scalp EEG, further investigations using intracranial electrodes or combined imaging techniques are warranted to confirm these abnormalities.

## Conclusions

In conclusion, this study highlights the significance of increased theta power and connectivity in mTLE-HS patients, indicating their important role in the epileptogenic network. Our findings suggest that higher theta power in mTLE-HS patients is associated with a more severe degree of epilepsy, presenting this abnormality within the lesion as well as in other more widespread regions due to the network properties of the brain. Additionally, a positive correlation was observed between theta power in the contralateral temporal lobe and the seizure frequency, supporting the hypothesis of lateralized buildup of rhythmic seizure activity. Furthermore, our results illustrate a positive correlation between theta power and the number of ASMs used, which may be related to the neurotoxic effects. Notably, third-generation ASMs showed fewer adverse effects on slow activity of the brain. Moreover, our results provide evidence for stronger neural connections from the temporal lobe to the parietal lobe (as well as the surrounding central regions), which may contribute to cognitive impairments in mTLE-HS patients. These findings may provide a new reference for alleviating cognitive disorders such as language processing and executive functions in this patient population.

## Supplementary Information

Supplementary Material 1.

## Data Availability

The datasets used and analyzed during the current study are available from the corresponding author on reasonable request.

## Abbreviations

ASMs: Anti-seizure medications
DRE: Drug-resistant epilepsy
EEG: Electroencephalogram
HCs: Healthy controls
HS: Hippocampal sclerosis
HS-L: The left HS group
HS-R: The right HS group
LCM: Lacosamide
MRI: Magnetic resonance imaging
mTLE-HS: Mesial temporal lobe epilepsy with hippocampal sclerosis
NHS3: The National Hospital Seizure Severity Scale
PER: Perampanel
PLV: Phase-locking value
PSD: Power spectral density
ROI: Regions of interest
TLE: Temporal lobe epilepsy
VEEG: Video electroencephalography
ZNS: Zonisamide
