# Supplementary material for: Differences and potential mechanisms of theta oscillation and temporoparietal and temporal-central networks in temporal lobe epilepsy patients with unilateral hippocampal sclerosis
Source: Acta Epileptol. 2024 Aug 25;6:26. doi: 10.1186/s42494-024-00170-7 (PMC11960361; doi:10.1186/s42494-024-00170-7)
Supplement: Supplementary file 1 — Supplementary Material 1. [file 42494_2024_170_MOESM1_ESM.docx]

Supplementary materials

**Supplementary Table 1.** Correlation between PSD in theta band and clinical data in the mTLE-HS group

| Characteristics | T3 | | T4 | | P3 | | C3 | | C4 | | Cz | | | FC4 | | |
| --- | --- | --- | --- | --- | --- | --- | --- | --- | --- | --- | --- | --- | --- | --- | --- | --- |
|  | *P* value | *R* value | *P* value | *R* value | *P* value | *R* value | *P* value | *R* value | *P* value | *R* value | *P* value | *R* value | *P* value | | *R* value |  |
| Age | 0.14 | -0.45 | 0.14 | -0.33 | 0.14 | -0.35 | 0.14 | -0.32 | 0.14 | -0.34 | 0.14 | -0.34 | 0.14 | | -0.38 |  |
| Epilepsy duration | 0.98 | -0.21 | 0.98 | 0.01 | 0.98 | 0.09 | 0.98 | 0.04 | 0.98 | 0.08 | 0.98 | -0.01 | 0.98 | | -0.06 |  |
| Drug-resistant epilepsy (DER) | 0.65 | 0.31 | 0.97 | 0.01 | 0.65 | 0.13 | 0.65 | 0.20 | 0.65 | 0.13 | 0.65 | 0.16 | 0.65 | | 0.27 |  |
| Seizure freedom | 0.96 | -0.05 | 0.96 | 0.01 | 0.96 | -0.03 | 0.96 | -0.01 | 0.96 | 0.04 | 0.96 | -0.09 | 0.96 | | -0.17 |  |
| Seizure frequency (seizure days/year) | 0.59 | 0.12 | 0.02 | 0.64 | 0.17 | 0.40 | 0.17 | 0.36 | 0.17 | 0.33 | 0.17 | 0.40 | 0.17 | | 0.33 |  |
| Number of ASMs | 0.18 | 0.30 | 0.08 | 0.39 | 0.02 | 0.53 | 0.02 | 0.57 | 0.02 | 0.52 | 0.02 | 0.61 | 0.04 | | 0.47 |  |
| Third-generation ASMs | 0.25 | 0.40 | 0.67 | 0.09 | 0.25 | 0.29 | 0.25 | 0.36 | 0.25 | 0.31 | 0.25 | 0.34 | 0.32 | | 0.24 |  |
| NHS3 sores | 0.20 | 0.29 | 0.01 | 0.66 | 0.02 | 0.57 | 0.04 | 0.47 | 0.04 | 0.46 | 0.04 | 0.49 | 0.09 | | 0.39 |  |

**P* < 0.05, ***P* < 0.01, FDR-corrected.
